# The Specific Mechanism of TREM2 Regulation of Synaptic Clearance in Alzheimer’s Disease

**DOI:** 10.3389/fimmu.2022.845897

**Published:** 2022-05-19

**Authors:** Qi Qin, Meng Wang, Yunsi Yin, Yi Tang

**Affiliations:** ^1^Innovation Center for Neurological Disorders, Department of Neurology, Xuanwu Hospital, Capital Medical University, Beijing, China; ^2^National Center for Neurological Disorders, National Clinical Research Center for Geriatric Diseases, Beijing, China

**Keywords:** Alzheimer disease, synaptic clearance, microglia, TREM2 (triggering receptor expressed on myeloid cells), APOE, complement

## Abstract

Alzheimer’s disease (AD) is a progressive neurodegenerative disease. Synaptic dysfunction is an integral feature of AD pathophysiology and a significant factor in early cognitive impairment in AD. Microglia, which are intrinsic immune cells in the central nervous system, play important regulatory roles in the process of synapse formation. Microglia can refine synaptic connections through synaptic clearance to ensure accurate synaptic transmission. Synaptic clearance is not only existed during central nervous system development but also aberrantly activated during AD pathology. However, the mechanisms of synaptic clearance in AD remain to be investigated. TREM2 is involved in the synaptic clearance of microglia, acting alone or with other molecules, such as apolipoprotein E (APOE). In addition, C1q is essential for microglia-mediated synaptic clearance. In this review, we systematically summarized the potential mechanisms of microglia involved in synaptic clearance, comprehensively reviewed the role of TREM2 in microglia regulating synaptic clearance and proposed our hypothesis that TREM2 interacts with APOE and C1q to promote synaptic clearance. This review provides new insights into the role of TREM2 regulation in microglia synaptic clearance and provides potential prospects for the treatment of AD.

## Introduction

Alzheimer’s disease (AD) is an age-related neurodegenerative disorder characterized by progressive memory loss and cognitive dysfunction (1). The typical pathological changes in AD are neuronal plaques and neurofibrillary tangles (2). Oligomeric amyloid-beta peptide (Aβ) has been shown to play an important role in cognitive impairment in AD. The primary targets of Aβ are synapses and synapse-related signaling pathways, which damage synaptic plasticity, learning and memory (3). Another key component of AD pathogenesis is tau protein hyperphosphorylation to form neurofibrillary tangles (NFTs). NFTs are less susceptible to degradation by proteolytic enzymes and accumulate in neurons, disrupting the normal function of neuronal axons, leading to structural and functional abnormalities of synapses, interfering with synaptic transmission between neurons, and ultimately leading to neurodegeneration (4). Thus, synaptic loss and altered synaptic plasticity are the neurobiological basis of cognitive impairment in Alzheimer’s disease, and synaptic damage is a manifestation of AD pathological impairment.

Synapses are the key sites of functional connections and information transfer between neurons, and synaptic plasticity is the cellular and morphological basis of learning and memory (5, 6). Synaptic plasticity is thought to play key roles in the development of neural circuitry and impairments in synaptic plasticity contribute to several prominent neuropsychiatric disorders, such as AD (7). Synapse clearance, also known as synaptic elimination, can eliminate unnecessary synapses and keep the morphological and functional maturation of the remaining synapses, has been thought to be a critical step in neural circuits stability and an essential mechanism underlying synaptic plasticity in the central nervous system (CNS) (8). Mounting evidence indicates that microglia plays an important role in synapse clearance (9).

Microglia are resident immune cells in the CNS that regulate brain development, maintenance of neuronal networks, and injury repair (10). Microglia serve as brain macrophages. They are responsible for the elimination of protein aggregates, redundant synapses, and other particulate and soluble antigens that may endanger the CNS. Microglia functional changes are implicated in brain development and aging, as well as in neurodegeneration (11). Studies have shown that normal microglia phagocytose and clear toxic Aβ oligomers, preventing the development of AD (12). However, excessive Aβ oligomers can stimulate the complement cascade pathway and induce microglia to excessively phagocytose and clear synapses in a manner similar to normal synaptic pruning, resulting in synaptic loss in AD patients (13). Overall, microglia can mediate synapse loss in AD progression. However, the mechanisms of microglial regulation remain to be investigated.

Triggering receptor expressed on myeloid cells 2 (TREM2) is an AD risk gene identified in recent years, and mutations in its coding region significantly increase the risk of AD (14, 15). TREM2 is mainly expressed by microglia in the nervous system and regulates microglial activation, proliferation, survival, phagocytosis, and other biological functions. Studies have shown that TREM2 is essential for synaptic clearance (16), but the specific mechanism by which TREM2 regulates synaptic clearance still needs to be explored.

Apolipoprotein E (APOE), the strongest genetic risk factor for AD, regulates neurodegeneration mainly by modulating microglial activation (17). APOE is synthesized and secreted mainly by astrocytes in the activated state, but in AD, the level of APOE expression in plaque-associated microglia is significantly increased and strongly induced in a TREM2-dependent manner (17, 18). Emerging evidence suggests that APOE binds to TREM2 and APOE are putative ligands for TREM2 (19), thus raising the possibility of an APOE-TREM2 interaction may drive the transcriptional phenotype of dysfunctional microglia and modulate AD pathology (20). However, whether APOE-TREM2 pathway participate in microglia-induced synaptic clearance needs further investigation.

Recent findings suggest that the classic complement cascade mediates CNS synaptic clearance (21, 22). The complement proteins C1q and C3 are involved in synaptic clearance by microglia (23). During central nervous system development, redundant neuronal synapses express the complement protein C1q, which is the initiator protein of the classic complement cascade and activates CR3 receptors, and microglia are the only cells in the brain that can express CR3 receptors (24). Activation of the complement signaling pathway allows microglia to recognize synapses, leading to synaptic clearance (25). Inhibition of C1q, C3 or the microglial complement receptor CR3 reduces microglial phagocytosis and prevent synaptic loss (26).

In this review, we systematically summarized the possible mechanisms of microglia involved in synaptic clearance in AD, comprehensively reviewed the role of TREM2 in microglia regulating synaptic clearance and explore the relationship of TREM2, APOE and C1q in synaptic clearance of microglia. We proposed our hypothesis that TREM2 interacts with APOE and C1q to promote synaptic clearance and impaired synaptic plasticity in AD. This review provides new insights into the role of TREM2 regulation in microglia synaptic clearance and provides potential prospects for the treatment of AD.

## Role of Microglia in Synaptic Clearance

### Microglia and Synapses in AD

Neurons in the brain rapidly extend their axons and dendrites after birth and form cellular connections between neurons (27). Synapses are the key sites of neuronal information transmission and play an irreplaceable role in the CNS (7). Synaptic loss is one of the important histological changes in AD patients, and the degree of synaptic loss correlates with the degree of dementia in AD patients (28–30).

Recent evidence shows that microglia can create tight connections with neuronal synapses *in vivo* and continually contact synapses in an activity and experience dependent manner, and play a functionally dynamic role in synaptic plasticity (31). Microglia can also contribute to structural plasticity through the elimination of synapses *via* phagocytic mechanisms, which is necessary for normal cognition (11). Based on these evidence, we suggest that microglia is essential for normal synaptic and structural plasticity that supports cognition (32). While in AD condition, plenty of evidence show microglial interactions with synapses obviously affect the maturation of synapses and neuronal viability (4, 31). For example, previous studies have shown that the accumulation of soluble Aβ activates microglia in the brain to release proinflammatory factors that mediate synaptic dysfunction (3). Activated microglia also clear synapses and promote synaptic remodeling (9).

In a word, synaptic dysfunction is a key initiator of AD pathology. Elucidating the underlying molecular mechanisms of microglia-synapse pathways may provide further insights into immune system regulation of neuronal circuit development in the AD brain and novel approaches for AD treatment.

### Microglia-Mediated Synaptic Clearance

In the last few years, plenty studies have shown that microglia can mediate synaptic clearance. The pathway of complement-mediated synaptic clearance has been extensively studied (33). Synapses requiring pruning can be localized and labelled by classic complement component 1q (C1q) and phagocytosed by complement receptor 3 (CR3)-mediated microglia (34, 35). Functional blockade of C1q by C1q antibodies or artificial knockdown of the C1q gene can reduce Aβ oligomer-induced and tau-induced excessive synaptic phagocytosis and improve synaptic deficits (36, 37). In addition, removal of the complement component C3 or knockdown of the CR3 gene also protected synaptic loss in the brains of AD transgenic mice (34, 38).

Besides complement system, TREM2 is also essential for microglia-mediated synaptic pruning (16). A lack of TREM2 receptors leads to impaired synaptic clearance, increased dendritic spine density and enhanced excitatory neurotransmission (39). Studies have shown that phosphatidylserine exposure at the synapse may be an “eat me” signal for TREM2 receptors (40). Therefore, TREM2 can mediate synaptic clearance by regulating microglia phagocytosis.

Another pathway through which microglia mediate synaptic clearance is the phagocytic signaling pathway of C-X3-C motile chemokine receptor 1 (CX3CR1) (41). In mice lacking Cx3cr1, microglia numbers were transiently reduced in the developing brain and synaptic pruning was delayed (42). Knockdown of CX3CL1 resulted in reduced social interactions and increased repetitive behavioral traits in both juvenile and adult mice (43). These results strongly suggest that microglia perform synaptic clearance *via* CX3CL1/CX3CR1 signaling.

In AD pathology, the abnormal mechanism of synaptic clearance mediated by microglia is not clear. Hence, we will summarize the various factors associated with microglia-mediated synaptic clearance in AD.

## The Mechanism May Involve in Microglia-Mediated Synaptic Clearance in AD

In recent years, genome-wide association studies (GWAS) have identified more than 25 genetic loci that are strongly associated with the risk of developing late-onset Alzheimer’s disease (LOAD), one of them is TREM2 gene variants (44). TREM2 has been suggested to play a crucial role in AD pathogenesis. TREM2 may act as a multifaceted player in microglial functions in AD brain homeostasis. TREM2 can not only influence microglial functions in amyloid and taupathologies, but also participate in inflammatory responses and metabolism. In this review, we will focus the role of TREM2 in microglia-mediated synaptic clearance in AD.

APOE is also undoubtedly associated with LOAD (45). AD patients carrying the ϵ4 allele of APOE have an earlier age of onset, more severe amyloid, tau deposition and brain atrophy, and more rapid disease progression (17). APOE not only involve in the aggregation and clearance of Aβ but also participate in the regulation of microglial activation, immune regulation and cytokine release (46). However, the role of APOE in synaptic clearance and neural pathway regulation needs to be further explored.

Furthermore, the complement system has recently been shown to plays both neuroprotective and neuroinflammatory roles in AD pathophysiology (47). During the early stages of AD, the complement system can succeed in clearing Aβ in conjunction with glial cells (48). However, when Aβ starts to accumulate and Aβ plaques develop, the complement system can further increase the phagocytic capability of microglia (49). Here, we will discuss how the complement system is involved in the effective functioning of microglia-mediated synaptic clearance in Alzheimer’s disease.

### TREM2 Is Involved in Synaptic Clearance by Microglia in AD

TREM2 is a transmembrane protein expressed in myeloid microglia and play important roles in functional maintenance of normal brain activity (50). Recent whole-genome sequencing studies have shown that rare TREM2 variants increase the risk of AD by 2-4-fold (15). In the cerebrospinal fluid (CSF) of AD patients, soluble TREM2 has been shown to correlate with total tau and phosphorylated tau (p-tau, Thr181) levels (51). Therefore, TREM2 has become a new hotspot in the study of AD pathogenesis and treatment.

TREM2 regulates a variety of biological functions (52–54), but the molecular mechanisms involved in AD pathogenesis are unclear. Most of the current studies on TREM2 regulation of microglial function have focused on its role in the regulation of abnormal protein accumulation and neuroinflammation (55–57). For example, TREM2 has been shown to have a critical role in reducing Aβ-induced neuronal atrophy and tau seeding/spreading (55, 56, 58). Other studies also point out TREM2 may affect the inflammatory response and energy metabolism, regulating microglial phagocytosis of abnormal proteins and damaged neurons (50, 52).

It is worth mentioning that several recent studies suggest that TREM2 is also essential for synaptic clearance by microglia (16, 39, 59). *In vitro* experiments showed that TREM2 knockdown significantly affected microglial activation and phagocytosis. *In vivo*, TREM2 mutant mice showed abnormal microglial synaptic phagocytosis, and functional magnetic resonance imaging also revealed altered functional connectivity in neural pathways throughout the brain (16). In TREM2-deficient mice, microglia fail to recognize redundant synapses, synaptic modification is absent, neuronal synaptic density is increased, arousal is enhanced, normal brain function is impaired, and the mice develop abnormal behaviors and social deficits (39). All in all, these data suggest microglia modified synapses *via* phagocytosis, which required the involvement of TREM2.

### APOE Interacts With TREM2 in AD

Both APOE and TREM2 were identified as independent risk factors for LOAD. They are also expressed in glia cells and related immune response. Both TREM2 and APOE play important roles in promoting microglial activation, survival, and barrier formation. TREM2 expression and function are positively associated with APOE expression in AD pathology (60), and APOE expression may be dependent on TREM2 regulation (18). Several groups have shown that TREM2 binds to APOE (61–63). APOE can increase the phagocytosis of apoptotic neurons *via* the TREM2 pathway. The TREM2 R47H variant was shown to reduce TREM2 affinity to bind APOE. Besides, recent study suggests that human APOE contain the binding site for TREM2, and that there is an APOE-isoform-dependent binding to TREM2. Recent reports also identified TREM2-APOE pathway can drive the transcriptional phenotype of dysfunctional microglia in AD, which can induce a shift from a homeostatic phenotype to a neurodegenerative phenotype in microglia (62).

Overall, emerging evidence suggests that APOE binds to TREM2 and APOE are putative ligands for TREM2, thus raising the possibility of an APOE-TREM2 interaction modulating different aspects of AD pathology. TREM2-APOE interaction may promote microglial activation, survival, phagocytosis and thus modulate inflammatory responses and neuronal injury. However, whether the TREM2-APOE pathway can modulate microglial synaptic clearance remains unclear and needs to be further investigated.

### Complement Signaling Pathway Involves in Microglia-Mediated Synaptic Clearance

Previous studies have identified the complement signaling pathway as a component of the innate immune system that is involved in synaptic clearance by microglia in AD (21, 47). C1q, the initiating protein in the classical complement cascade, is expressed by postnatal neurons in response to immature astrocytes and is localized at synapses (64). During CNS development, redundant neuronal synapses express the complement protein C1q, which activates CR3 receptors, and since microglia are the only cells that can express CR3 in the brain, activation of the complement signaling pathway enables the recognition of synapses by microglia, leading to phagocytosis and synaptic clearance. Mice lacking either complement protein C1q or downstream complement protein C3 exhibit a greater sustained deficit in CNS synaptic elimination (21, 24). Hong et al. examined a mouse model of Alzheimer’s disease and showed that C1q was significantly increased before significant plaque deposition and was associated with synapse clearance (65). Inhibition of C1q, C3, or the complement receptor CR3 in microglia reduces the number of phagocytic microglia and decreases the extent of synaptic loss in AD.

C1q and APOE were previously thought to act separately and perform independent tasks in many tissue contexts. Recent studies have shown a correlation between C1q and APOE (66). Currently, C1q has been shown to colocalize with APOE in the brain in human AD (67). *In vitro* experiments also demonstrated that APOE is a checkpoint inhibitor of classic complement cascade activity (68). All human APOE isoforms attenuate classic complement cascade activity *in vitro* through high affinity binding to activated C1q. The C1q-APOE complex is a marker of sustained complement activity in Aβ plaques *in vivo*. The C1q-APOE complex in Aβ plaques is associated with reduced cognitive performance. APOE activates C1q through the formation of the C1q-APOE complex, which in turn initiates the classic complement cascade. In AD, Aβ deposits may lead to the loss of synapses by activating complement-related clearance pathways. In summary, APOE binds to C1q and regulates the activation of the classic complement cascade. C1q-APOE complex may affect AD by regulating synaptic clearance by microglia.

Moreover, in recent years, it has been found that the levels of both TREM2 and C1q are significantly increased around Aβ, suggesting an association between TREM2 and C1q in microglia-mediated synaptic phagocytosis (40, 64). Therefore, clarifying the interaction between TREM2 and C1q in microglia-mediated synaptic clearance will further refine our understanding of TREM2 and the complement system in AD.

In conclusion, the exact relationship between TREM2, APOE and C1q is still unclear. Therefore, further in-depth and comprehensive studies are needed to investigate how these three pathways regulate microglial synaptic clearance in AD **Table 1**.

**Table 1:** The specific mechanism in microglia-mediated synaptic clearance in Alzheimer’s Disease.

Factors	Mechanism in synaptic clearance in Alzheimer’s Disease
TREM2	1) Lack of TREM2 receptors lead to impaired synaptic clearance. 2)TREM2 knockdown significantly affect microglial activation and phagocytosis. 3) TREM2 recognize phosphatidylserine “eat me” signal at the synapses.
C1q	1) Synapses requiring pruning can be localized and labelled by C1q. 2) Inhibition of C1q can reduce Aβ oligomer-induced and tau-induced excessive synaptic phagocytosis and improve synaptic deficits. 3) Removal of the complement component protect synaptic loss in the brains of AD transgenic mice.
CX3CR1	1) In mice lacking Cx3cr1, synaptic pruning is delayed. 2) Knockdown of CX3CL1 results in reduced social interactions and increases repetitive behavioral traits.
APOE	1) APOE can increase the phagocytosis of apoptotic neurons *via* the TREM2 pathway. 2) APOE is a checkpoint inhibitor of classic complement cascade activity.
TREM2-APOE	1) APOE binds to TREM2 and APOE are putative ligands for TREM2 2) TREM2-APOE pathway can drive the transcriptional phenotype of dysfunctional microglia in AD. 3) TREM2-APOE interaction promotes microglial activation, survival, phagocytosis.
APOE-C1q	1) C1q has been shown to colocalize with APOE in the brain in human AD. 2) APOE activates C1q through the formation of the C1q-APOE complex, which in turn initiates the classic complement cascade. 3) C1q-APOE complex affect microglia-mediated synaptic clearance.
TREM2-C1q	An association between TREM2 and C1q in microglia-mediated synaptic phagocytosis needs to be explored.

## Summary and Prospects

**Figure 1:**
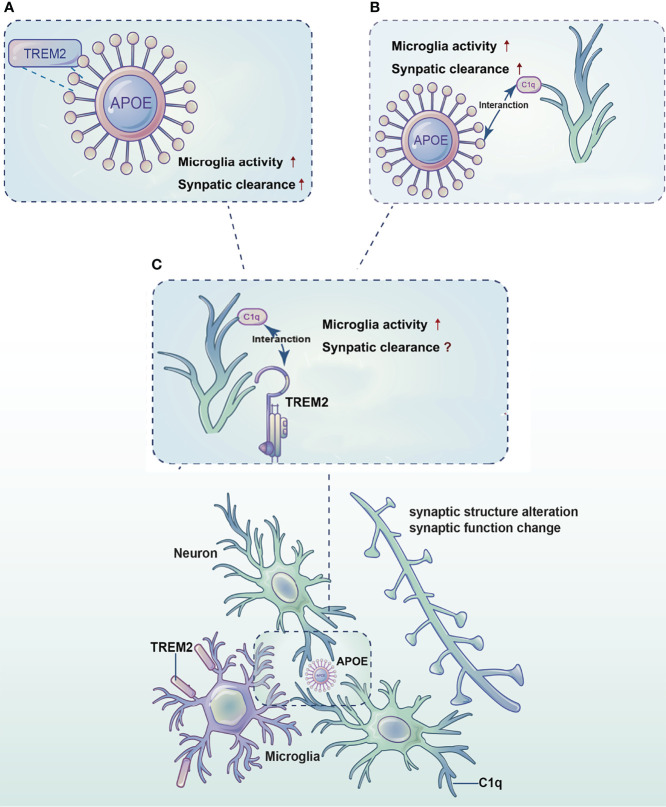
Diagram of the synergistic effects of TREM2, APOE and C1q in microglia-mediated synaptic clearance in AD. TREM2, APOE and C1q interact with each other and promote microglia mediated synaptic clearance in AD. **(A)** TREM2 binds to APOE and promotes microglia activation and survival, thereby regulates synaptic clearance. **(B)** APOE activates C1q by forming the C1q-APOE complex, which in turn initiates the classic complement cascade to regulate synaptic clearance by microglia. **(C)** An association between TREM2 and the complement protein C1q needs to be explored.

As the most important immune cells in the brain, microglia play significant roles in almost all central nervous system disease processes. The studies of microglia function have been a hot topic in the field of AD research. Microglia can refine synaptic connections through phagocytosis to ensure accurate transmission of information. TREM2, a microglia surface receptor, is essential for synaptic clearance by microglia, but the exact mechanism needs to be further explored. TREM2 binds with high affinity to APOE and promotes the clearance of apoptotic neurons and cellular debris by microglia. Previous studies have identified the complement signaling pathway is also involved in synaptic clearance. APOE may affect the classic complement cascade through the C1q-APOE complex, which affects microglia and influences synaptic pruning. In recent years, significantly increased levels of both TREM2 and the complement protein C1q have been found around Aβ, suggesting an association between TREM2 and C1q. Therefore, we propose that TREM2, APOE and C1q interact with each other and promote microglia mediated synaptic clearance in AD **Figure 1**.

In this review, we systematically summarized the potential mechanisms of microglia involved in synaptic clearance, comprehensively reviewed the role of TREM2 in microglia regulating synaptic clearance and proposed our hypothesis that TREM2 interacts with APOE and C1q to promote synaptic clearance in AD. This review provides new insights into the role of TREM2 regulation in microglia synaptic clearance and provides new clues to elucidate the role of TREM2 in the pathogenesis of AD.

## Author Contributions

QQ: Conceptualization, Writing - review and editing, Supervision. MW: Conceptualization, Writing - original draft, Writing - review and editing. YY: Writing - original draft. YT: Conceptualization, Writing - review and editing, Supervision, Project administration. All authors read and approved the final manuscript.

## Funding

This work was supported by Capital’s Funds for HealthImprovement and Research [grant number CFH 2020-4-1033]; Beijing NOVA Program [grant number Z211100002121051];Beijing Hospitals Authority Innovation Studio of Young Staff Funding Support [grant number 202118] and Young Elite Scientists Sponsorship Program by CAST [grant number 2021QNRC001].

## Conflict of Interest

The authors declare that the research was conducted in the absence of any commercial or financial relationships that could be construed as a potential conflict of interest.

## Publisher’s Note

All claims expressed in this article are solely those of the authors and do not necessarily represent those of their affiliated organizations, or those of the publisher, the editors and the reviewers. Any product that may be evaluated in this article, or claim that may be made by its manufacturer, is not guaranteed or endorsed by the publisher.

## Abbreviations

AD, Alzheimer’s disease; APOE, apolipoprotein E; Aβ, amyloid-β; C1q:complement component 1q; CNS, central nervous system; CR3:complement receptor 3; CSF, cerebrospinal fluid; GWAS, Genome-wide association studies; LOAD, late-onset AD; NFTs, neurofibrillary tangles; TREM2, triggering receptor expressed on myeloid cells 2.
